# Development and characterization of a recombinant, hypoallergenic, peptide-based vaccine for grass pollen allergy

**DOI:** 10.1016/j.jaci.2014.09.012

**Published:** 2015-05

**Authors:** Margarete Focke-Tejkl, Milena Weber, Katarzyna Niespodziana, Angela Neubauer, Hans Huber, Rainer Henning, Gottfried Stegfellner, Bernhard Maderegger, Martina Hauer, Frank Stolz, Verena Niederberger, Katharina Marth, Julia Eckl-Dorna, Richard Weiss, Josef Thalhamer, Katharina Blatt, Peter Valent, Rudolf Valenta

**Affiliations:** a Division of Immunopathology, Department of Pathophysiology and Allergy Research, Center of Pathophysiology, Infectiology and Immunology, Medical University of Vienna, Vienna, Austria; c Department of Otorhinolaryngology, Medical University of Vienna, Vienna, Austria; e Department of Internal Medicine I, Division of Hematology & Hemostaseology, Medical University of Vienna, Vienna, Austria; b Biomay AG, Vienna, Austria; d Department of Molecular Biology, Division of Allergy and Immunology, University of Salzburg, Salzburg, Austria

**Keywords:** Grass pollen allergy, allergen, recombinant allergen, recombinant hypoallergenic allergen derivative, allergen-specific immunotherapy, peptide-carrier technology, CFA, Complete Freund adjuvant, KLH, Keyhole limpet hemocyanin, SIT, Allergen-specific immunotherapy

## Abstract

**Background:**

Grass pollen is one of the most important sources of respiratory allergies worldwide.

**Objective:**

This study describes the development of a grass pollen allergy vaccine based on recombinant hypoallergenic derivatives of the major timothy grass pollen allergens Phl p 1, Phl p 2, Phl p 5, and Phl p 6 by using a peptide-carrier approach.

**Methods:**

Fusion proteins consisting of nonallergenic peptides from the 4 major timothy grass pollen allergens and the PreS protein from hepatitis B virus as a carrier were expressed in *Escherichia coli* and purified by means of chromatography. Recombinant PreS fusion proteins were tested for allergenic activity and T-cell activation by means of IgE serology, basophil activation testing, T-cell proliferation assays, and xMAP Luminex technology in patients with grass pollen allergy. Rabbits were immunized with PreS fusion proteins to characterize their immunogenicity.

**Results:**

Ten hypoallergenic PreS fusion proteins were constructed, expressed, and purified. According to immunogenicity and induction of allergen-specific blocking IgG antibodies, 4 hypoallergenic fusion proteins (BM321, BM322, BM325, and BM326) representing Phl p 1, Phl p 2, Phl p 5, and Phl p 6 were included as components in the vaccine termed BM32. BM321, BM322, BM325, and BM326 showed almost completely abolished allergenic activity and induced significantly reduced T-cell proliferation and release of proinflammatory cytokines in patients' PBMCs compared with grass pollen allergens. On immunization, they induced allergen-specific IgG antibodies, which inhibited patients' IgE binding to all 4 major allergens of grass pollen, as well as allergen-induced basophil activation.

**Conclusion:**

A recombinant hypoallergenic grass pollen allergy vaccine (BM32) consisting of 4 recombinant PreS-fused grass pollen allergen peptides was developed for safe immunotherapy of grass pollen allergy.

Because of their worldwide distribution and heavy pollen production, grasses are among the most important allergen sources.^1,2^ Grass pollen allergy affects almost 50% of patients, with IgE-associated allergy and allergic rhinoconjunctivitis (ie, hay fever) and asthma being the most frequent symptoms.^3,4^ Already in 1880, grass pollen was recognized as a potent allergen source, and provocation tests were developed for diagnosis of grass pollen allergy.^5^ In 1911, the first allergen-specific immunotherapy (SIT) trial was performed in patients with grass pollen allergy.^6^ SIT is based on the administration of the disease-eliciting allergens, with the goal to induce clinical tolerance in patients.^7^ It is the only allergen-specific treatment for allergic patients and, unlike pharmacotherapy, has disease-modifying and long-lasting effects.^8,9^ However, side effects caused by allergen administration limit the broad application of SIT for allergy treatment.^10^ The most frequent and severe side effects were found in SIT for grass pollen allergy when compared with that for other allergens.^11^

At present, several alternative forms of SIT are being developed and subjected to clinical evaluation to improve the safety, efficacy, and convenience of SIT. They include various routes of administration and molecular forms of treatment based on recombinant allergens, hypoallergenic allergen derivatives, and allergen-derived peptides.^12-23^

The spectrum of grass pollen allergens, in particular those from timothy grass pollen, which contains the majority of the relevant epitopes needed for grass pollen SIT,^24^ has been well defined.^2^ In particular, those major timothy grass pollen allergens required as essential components for grass pollen SIT have been identified by using several serologic and clinical studies, and available data suggest that a mix of recombinant group 1, 2, 5, and 6 allergens is effective for treatment.^25-29^

Here we report the development and preclinical characterization of a hypoallergenic grass pollen allergy vaccine, designated BM32. The active components of the vaccine included nonallergenic peptides derived from the major IgE-binding sites of the 4 major timothy grass pollen allergens Phl p 1, Phl p 2, Phl p 5, and Phl p 6, which were fused to the hepatitis B–derived PreS domain and expressed in *Escherichia coli* in large amounts and purified to homogeneity. In the fusion proteins the PreS domain functions as a nonallergenic carrier protein, which provides T-cell help for the induction of allergen-specific blocking antibodies on immunization.

The reduction of allergenic and inflammatory activity of the fusion proteins was investigated by using IgE reactivity and basophil activation testing, as well as by studying T-cell proliferation and cytokine release in allergic patients. Furthermore, the fusion proteins were formulated as a vaccine through adsorption to aluminum hydroxide and, on immunization of rabbits or treatment of sensitized mice, induced allergen-specific IgG antibodies, which blocked IgE binding to the allergens and allergen-specific basophil activation in allergic patients.

## Methods

### Allergic patients, timothy grass pollen extract, recombinant allergens, synthetic peptides, and antibodies

Information regarding materials and patients can be found in the Methods section in this article's Online Repository at www.jacionline.org.

### Expression and purification of hexahistidine-tagged recombinant PreS fusion proteins and recombinant PreS

Codon-optimized DNAs encoding fusion proteins consisting of allergen-derived peptides fused to the N- and C-terminus of PreS and containing a C-terminal hexahistidine tag were synthesized (GenScript, Piscataway, NJ) and inserted into the *Nde*I/*Xho*I sites of plasmid pET-27b (Novagen, Darmstadt, Germany). Recombinant PreS and PreS fusion proteins were expressed in *E coli* strain BL21(DE3) (Stratagene, La Jolla, Calif). After induction of protein expression by adding isopropyl-β-D-thiogalactopyranoside to a final concentration of 0.7 mmol/L, cells were cultured for 3 hours at 37°C and then harvested by means of centrifugation at 3500 rpm for 10 minutes. His-tagged PreS and PreS fusion proteins were then purified by means of nickel affinity chromatography (see the Methods section in this article's Online Repository). Recombinant PreS fusion proteins without His-tags were also expressed in *E coli* and purified to homogeneity (H. Huber, unpublished data).

### Specificities and titers of specific rabbit antibodies and inhibition of IgE binding to allergens by rabbit antibodies in allergic patients

Reactivity and titers of rabbit antibodies specific for grass pollen allergens, allergen-derived peptides, and PreS were determined by means of ELISA.^30^ Inhibition of the binding of allergic patients' IgE to grass pollen allergens by rabbit antibodies was determined by using an IgE competition ELISA.^31^

### Assessment of IgE reactivity

IgE reactivity to allergens, peptides, PreS, and PreS fusion proteins in allergic patients was determined by using ELISA and/or IgE dot blotting with a RAST-based technique.^32^

### Determination of allergenic activity by measurement of allergen-induced upregulation of CD203c on patients' basophils

Heparinized blood samples obtained from patients with grass pollen allergy (n = 31) were incubated in triplicates with increasing concentrations of antigens (0.001-1 μg/mL), buffer, or the anti-IgE mAb E-124-2-8 (1 μg/mL; Immunotech, Marseille, France), and upregulation of CD203c expression was determined, as previously described.^33^ The inhibition of allergen-induced CD203c upregulation by specific IgG antibodies was studied, as previously described.^34^

### Lymphocyte proliferation and cytokine responses of PBMCs from patients with grass pollen allergy

PBMCs from patients with grass pollen allergy (n = 50) were isolated by means of Ficoll (Amersham Biosciences, Uppsala, Sweden) density gradient centrifugation. Aliquots of 2 × 10^5^ cells were resuspended in 200 μL of serum-free UltraCULTURE medium (BioWhittaker, Rockland, Me) supplemented with 2 mmol/L l-glutamine (Sigma, St Louis, Mo), 50 μmol/L β-mercaptoethanol (Sigma), and 0.02 mg of gentamicin per milliliter (Sigma) and stimulated with 3 concentrations (100 μg per well, 50 μg per well, and 25 μg per well) of timothy grass pollen extract from Stallergenes (Antony, France), with 3 concentrations (ie, 1 μg per well, 0.5 μg per well, or 0.25 μg per well of each of the 4 BM32 fusion proteins) of the BM32 fusion proteins, medium alone (negative control), or IL-2 (4 IE per well, positive control). The concentration of the major timothy grass pollen allergen Phl p 5 in the timothy grass pollen extract was determined with a quantitative sandwich ELISA^30^ to be 1 μg of Phl p 5 in 100 μg of total proteins. After 6 days of culture, proliferative responses were measured based on tritiated thymidine incorporation and were expressed as stimulation indices.^35^ IL-2, IL-4, IL-5, IL-6, IL-10, IL-12, IL-13, IFN-γ, TNF-α, and GM-CSF levels were quantified in supernatants from PBMC cultures, which were identically prepared as for the proliferation experiments by using the Bio-Plex Human 17-Plex Panel (Bio-Rad Laboratories, Hercules, Calif), xMAP Luminex fluorescent bead–based technology, and a Luminex 100 system for measurements (Luminex, Austin, Tex).^35^ Shown are results for 50 μg per well of timothy grass pollen extract and 0.5 μg per well of each BM32 protein.

### Murine model of grass pollen allergy and basophil activation testing in mice

Immunizations of mice with BM32 and measurement of T-cell and antibody responses were performed, as described in the Methods section in this article's Online Repository. A murine model of grass pollen allergy was established, treatment was performed with BM32 or PBS (control), and allergen-induced basophil activation was measured, as described in the Methods section in this article's Online Repository.

### Statistical analysis

For statistical analyses, GraphPad Prism 6 software (GraphPad Software, La Jolla, Calif) was used. Differences between the groups regarding T-cell reactivity are compared with the Wilcoxon signed rank test. Differences in basophil activation in mice were calculated by using the unpaired *t* test. *P* values of less than .05 were considered significant.

## Results

### Molecular design of the grass pollen allergy vaccine BM32 based on the peptide-carrier technology

**Fig 1:**
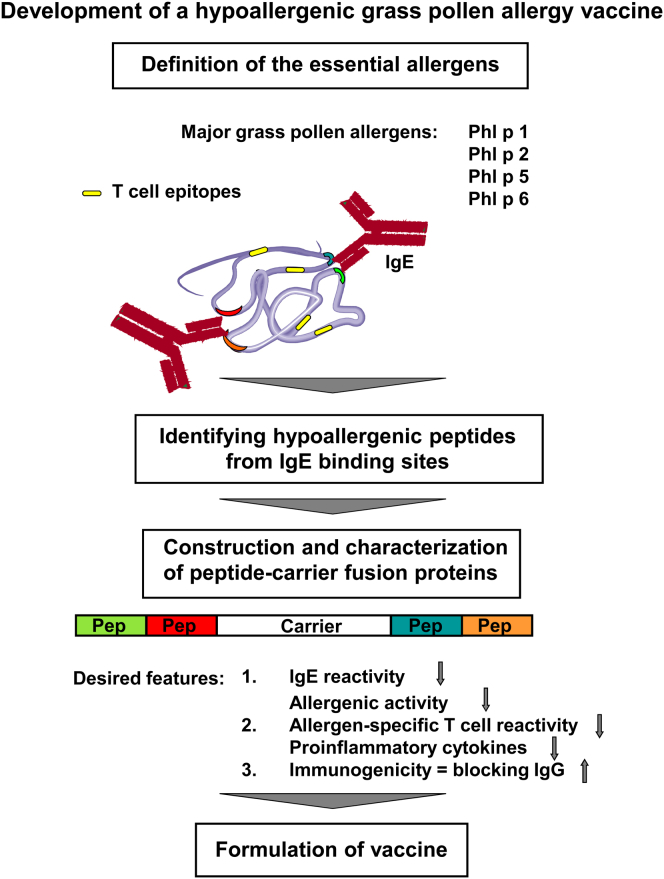
Scheme for the preclinical development of a hypoallergenic grass pollen allergy vaccine based on carrier-bound allergen peptides.

The design of the vaccine components of BM32 was carried out according to the process summarized in Fig 1. The first step in this process is the selection of the clinically relevant grass pollen allergens to be included. Phl p 1, Phl p 2, Phl p 5, and Phl p 6^25,26,36-39^ have been identified previously as essential vaccine components on the basis of IgE reactivity profiles of patients with grass pollen allergy, IgE cross-reactivity with natural grass pollen allergens, and assessments of allergenic activities. A mix of these wild-type allergens was tested clinically in an SIT study and was found to be efficacious.^27^

Protein design then began with identification of hypoallergenic peptides derived from the major IgE-binding sites of these allergens.^22,23^ Selection criteria were the absence of IgE binding, reduced potential to activate allergen-specific T cells, and induction of blocking antibody responses on immunization after conjugation to KLH. Suitable peptides had been already described for the 2 major allergens Phl p 1 and Phl p 5.^31,35^ In the case of Phl p 1, a C-terminal peptide, P5-1, was identified,^31,40^ whereas for Phl p 5, a mixture of 4 peptides (ie, P1-5, P2-5, P5-5, and P6-5) was found to be required to induce a sufficient blocking IgG antibody response.^35^

For identification of hypoallergenic Phl p 2 and Phl p 6 peptides, 4 Phl p 2–derived peptides (P1-2 to P4-2) and 4 Phl p 6–derived peptides (P1-6 to P4-6) were synthesized and tested.

**Fig E1:**
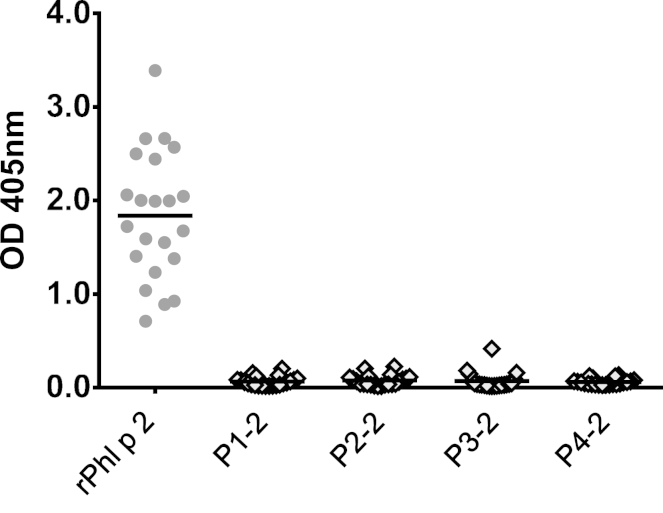
Comparison of IgE reactivity to rPhl p 2 and Phl p 2–derived peptides in patients. Displayed are IgE levels (*y-axis*: OD values; *horizontal bars* = medians) specific for Phl p 2 or Phl p 2–derived peptides (P1-2 to P4-2; *x-axis*) measured by means of ELISA in sera from patients with grass pollen allergy (n = 22).

Three nonallergenic peptides (see Table E1 in this article's Online Repository at www.jacionline.org [P1-2, P3-2, and P4-2]) had been identified for the construction of a hypoallergenic Phl p 2 mosaic protein.^41^ In addition to these 3 peptides, another peptide, P2-2, which, according to the nuclear magnetic resonance and crystal structure of Phl p 2 (1CQA PDB accession number),^42,43^ was rich in surface-exposed amino acids and which also contained several amino acids from a conformational epitope defined by an IgE Fab from an allergic patient on Phl p 2^43^ was synthesized. We tested the 4 Phl p 2–derived peptides for IgE reactivity and found that none of them showed any relevant IgE binding when compared with Phl p 2 (see Fig E1 in this article's Online Repository at www.jacionline.org).

**Fig E2:**
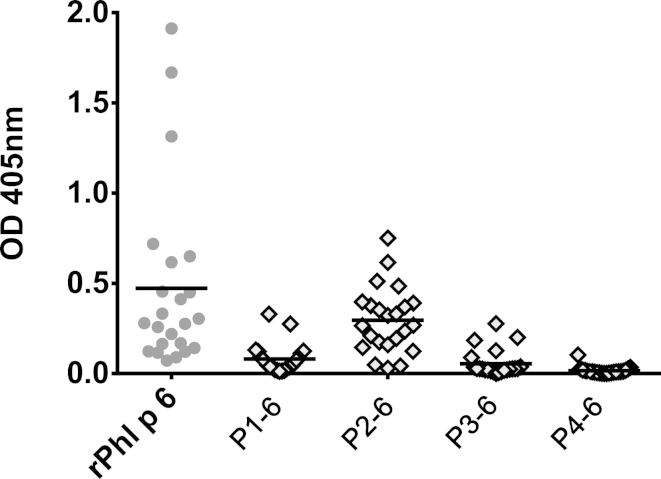
Comparison of IgE reactivity to rPhl p 6 and Phl p 6–derived peptides in patients. Displayed are IgE levels (*y-axis*: OD values; *horizontal bars* = medians) specific for Phl p 6 or Phl p 6–derived peptides (P1-6 to P4-6; *x-axis*) measured by means of ELISA in sera from patients with grass pollen allergy (n = 22).

The selection of peptides to be screened for IgE reactivity and immunogenicity of Phl p 6 was driven by several considerations. Phl p 6 consists of 4 anti-parallel α-helices.^39^ A recombinant Phl p 6 fragment comprising amino acids 31 to 110, thus lacking the N-terminal α-helix, was found to be hypoallergenic and to induce a robust Phl p 6–specific IgG response, inhibiting IgE binding to Phl p 6 in allergic patients.^39^ On the basis of this knowledge and computer-aided prediction of surface-exposed areas of Phl p 6, we synthesized 4 peptides (ie, P1-6 to P4-6; see Table E1), of which each roughly corresponded to the 4 α-helices of rPhl p 6.^39^ Only P2-6 showed IgE reactivity almost comparable with that of complete Phl p 6 (see Fig E2 in this article's Online Repository at www.jacionline.org).

**Fig E3:**
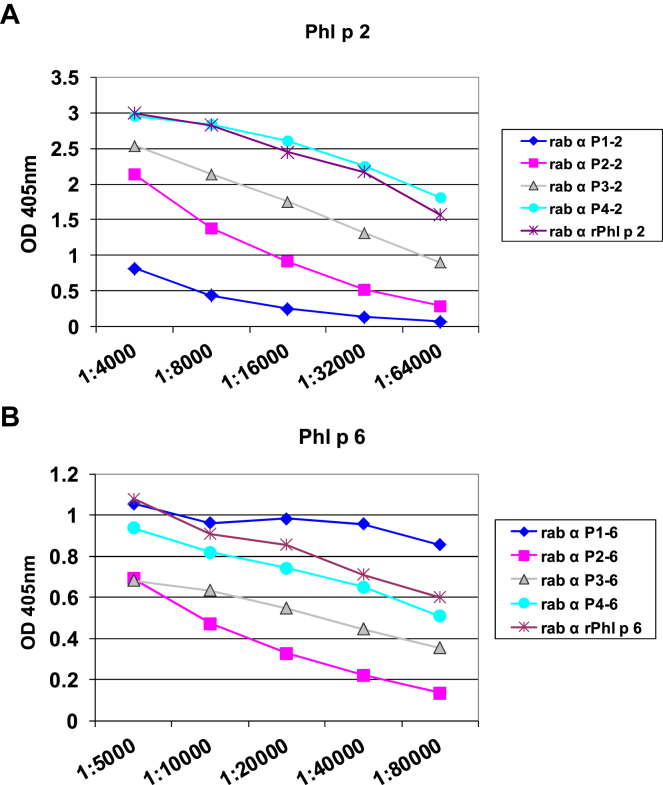
IgG reactivity of sera from peptide-immunized rabbits with Phl p 2 **(A)** and Phl p 6 **(B)**. IgG reactivities (mean ± SD OD levels of triplicates; *y-axes*) to Phl p 2 (Fig E3, *A*) or Phl p 6 (Fig E3, *B*) determined for different dilutions *(x-axes)* of sera from rabbits immunized with the complete allergens or KLH-coupled allergen-derived peptides are shown (Fig E3, *A*: Phl p 2 and Phl p 2 peptides P1-P4; Fig E3, *B*: Phl p 6 and Phl p 6 peptides P1-P4).

Next, we investigated which peptides induce allergen-specific IgG responses on immunization. Fig E3 in this article's Online Repository at www.jacionline.org shows reactivities of peptide-specific IgG antibodies with the corresponding allergens. For each of the 2 allergens, one peptide (ie, Phl p 2: P4-2; Phl p 6: P1-6) could be identified that induced levels of IgG antibodies comparable to those induced by the complete allergen (see Fig E3).

We then analyzed whether peptide-specific IgG antibodies inhibit IgE binding to the allergen in allergic patients. We found that anti–P4-2 (Phl p 2) and anti–P1-6 (Phl p 6) antibodies produced a greater than 70% mean inhibition of IgE binding to the corresponding allergens in allergic patients (see Tables E2 and E3 in this article's Online Repository at www.jacionline.org). Because these peptides also induced no relevant proliferation and proinflammatory cytokine responses in PBMCs from patients with grass pollen allergy (data not shown), they were selected as components to be included in the grass pollen vaccine.

**Fig 2:**
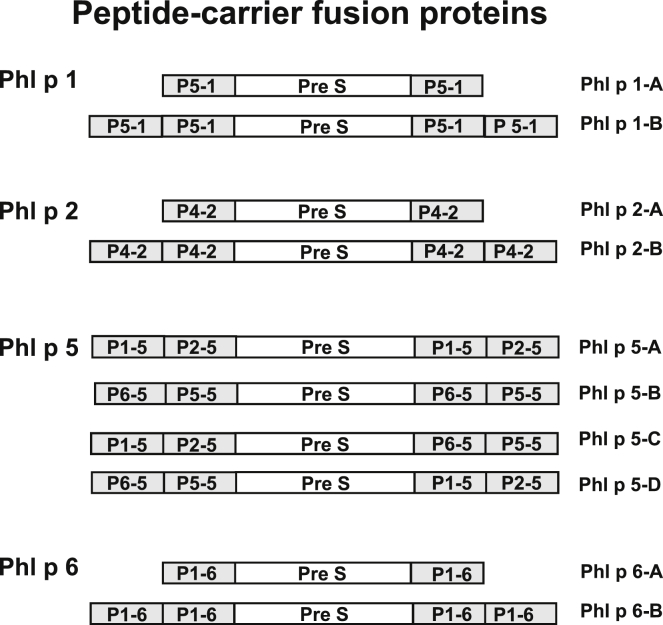
Overview of recombinant fusion proteins consisting of PreS-fused allergen peptides made for Phl p 1, Phl p 2, Phl p 5, and Phl p 6. Names of the fusion proteins are shown at the *right margin*.

An important feature of the peptide-carrier technology is that T-cell help for induction of allergen-specific IgG on immunization is obtained by fusing the peptides to a nonallergenic carrier molecule (Fig 1).^22,23,44^ The PreS coat protein derived from hepatitis B virus was selected based on its immunogenicity and safety in previous use as a hepatitis B vaccine and its high expression yield in *Escherichia coli*.^32,45,46^ Ten different PreS fusion proteins were designed by using peptides P5-1 of Phl p 1; P1-5, P2-5, P5-5, and P6-5 of Phl p 5; P4-2 of Phl p 2; and P1-6 of Phl p 6 as the allergen-specific moieties. They were expressed in *E coli*, purified, and characterized. A schematic representation of these fusion proteins is shown in Fig 2. For immunization experiments, the fusion proteins were adsorbed to aluminum hydroxide, which is one of the most common adjuvants used in SIT vaccines.

### Construction, expression, and purification of recombinant fusion proteins consisting of hepatitis B–derived PreS and allergen-derived peptides

For Phl p 1, Phl p 2, and Phl p 6, for which a single peptide was sufficient to induce allergen-specific blocking IgG, we engineered fusion proteins containing either 1 or 2 copies of the peptide fused to the N- and C-terminus of PreS, yielding fusion proteins with 2 (ie, Phl p 1-A, Phl p 2-A, and Phl p 6-A) or 4 (ie, Phl p 1-B, Phl p 2-B, Phl p 6-B) peptide copies (Fig 2). In the case of Phl p 5, 4 different peptides were required to induce blocking IgG. Therefore we constructed proteins containing P1-5 and P2-5 or P6-5 and P5-5 fused to the N-terminus, as well as the C-terminus, of PreS (Phl p 5-A and Phl p 5-B). In addition, 2 fusion proteins were made containing one copy of each of the 4 peptides, one in which P1-5 and P2-5 were added to the N-terminus and P6-5 and P5-5 were added to the C-terminus (ie, Phl p 5-C) and a second in which P1-5 and P2-5 were at the C-terminus and P6-5 and P5-5 were at the N-terminus (ie, Phl p 5-D; Fig 2).

**Fig E4:**
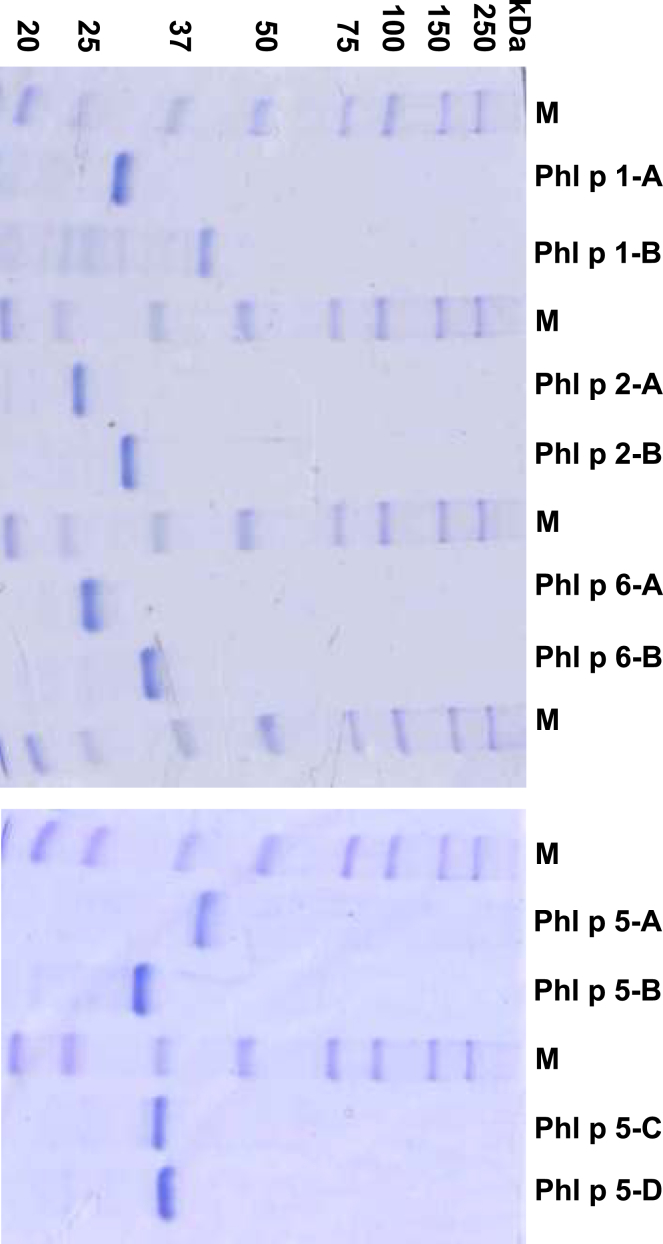
Coomassie-stained SDS-PAGE. Lanes contain purified fusion proteins or markers *(lanes M)*. Molecular weights (in kilodaltons) are indicated on the *left margin*.

The recombinant fusion proteins were expressed in *E coli* and purified as soluble proteins with good yields under conditions of laboratory scale expression (ie, 20-25 mg/L culture; see Fig E4 in this article's Online Repository at www.jacionline.org). Matrix-assisted laser desorption/ionization time-of-flight mass spectrometry of the recombinant fusion proteins showed that the molecular weights deduced from their sequences without methionines were in agreement with the actual masses (data not shown).

### Recombinant PreS fusion proteins show no IgE reactivity and induce allergen-specific IgG antibodies

**Fig 3:**
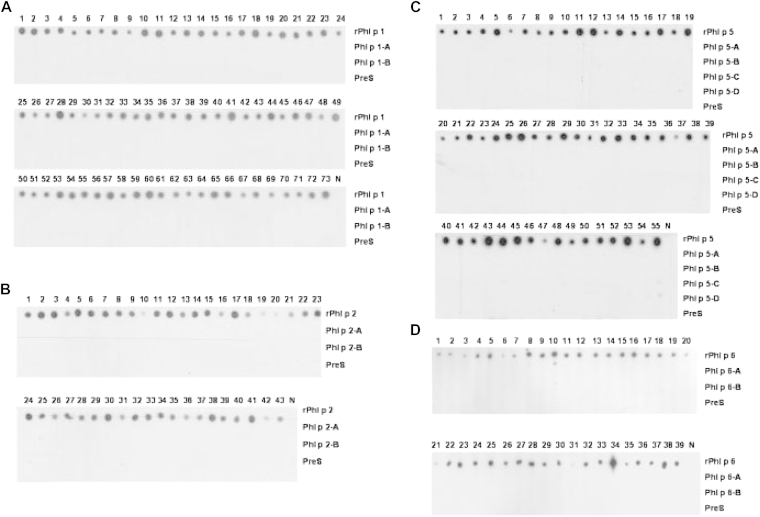
Comparison of the IgE reactivity of recombinant grass pollen allergens, fusion proteins, and PreS. IgE reactivity of sera from patients with grass pollen allergy and from a nonallergic person *(N)* to dot blots of rPhl p 1, Phl p 1-A, Phl p 1-B, and PreS **(A)**; rPhl p 2, Phl p 2-A, Phl p 2-B, and PreS **(B)**; rPhl p 5, Phl p 5-A, Phl p 5-B, Phl p 5-C, Phl p 5-D, and PreS **(C)**; and rPhl p 6, Phl p 6-A, Phl p 6-B, and PreS **(D)** are shown.

The recombinant fusion proteins were compared regarding IgE reactivity with the corresponding wild-type allergens in representative numbers of allergic patients (Phl p 1, n = 73; Phl p 2, n = 43; Phl p 5, n = 55; and Phl p 6, n = 39; Fig 3). None of the fusion proteins showed any detectable IgE reactivity, whereas each of the sera reacted with the wild-type allergens (Fig 3). Recombinant PreS showed no IgE reactivity, and sera from nonallergic subjects did not exhibit IgE reactivity with any of the proteins (Fig 3). The presence of the fusion proteins on the membrane was confirmed by testing with PreS and peptide-specific antibodies (data not shown).

To study the ability of the fusion proteins to induce blocking IgG antibodies against wild-type allergens on immunization, fusion proteins and, for comparison, complete allergens were adsorbed to complete Freund adjuvant (CFA) and incomplete Freund adjuvant or to aluminum hydroxide for immunization of rabbits.

**Fig E5:**
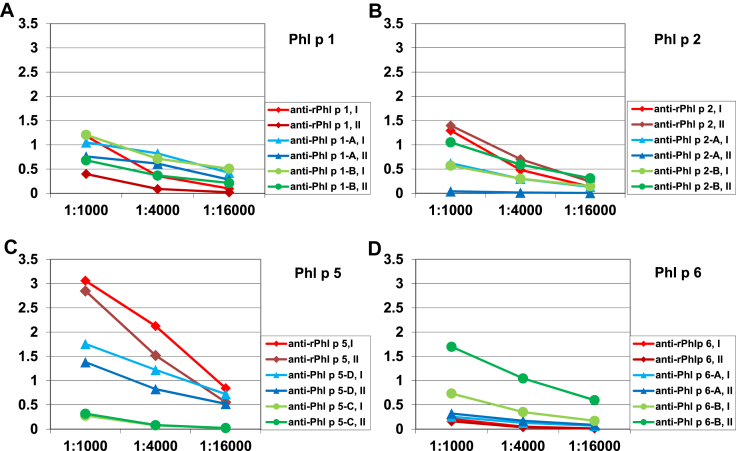
Allergen-specific IgG reactivity of rabbits immunized with aluminum hydroxide–adsorbed allergens or fusion proteins. IgG reactivities (*y-axes*, mean OD levels of triplicates) of rabbits immunized with the complete recombinant allergens or the fusion proteins to Phl p 1 **(A)**, Phl p 2 **(B)**, Phl p 5 **(C)**, or Phl p 6 **(D)** determined for different serum dilutions *(x-axes)* are shown. For each protein, 2 different rabbits were immunized (*I* and *II*).

The testing of different dilutions of the rabbit antisera for IgG reactivity to the wild-type allergens indicated that the fusion proteins containing 4 copies of the allergen-derived peptides induced higher titers of allergen-specific antibodies than fusion proteins containing only 2 peptide copies (see Fig E5 in this article's Online Repository at www.jacionline.org). Interestingly, Phl p 5-D, which contained P6-5-P5-5 at the N-terminus, induced higher Phl p 5–specific IgG levels than the fusion protein containing P6-5-P5-5 at the C-terminus (ie, Phl p 5-C; see Fig E5). The Phl p 5–specific IgG titers induced with Phl p 5-A or Phl p 5-B were not higher than those induced with Phl p 5-D (data not shown), which was then selected as a vaccine component because it combined all 4 Phl p 5 peptides in a single protein.

### Allergen-specific IgG antibodies induced by immunization with peptides and PreS fusion proteins inhibit allergic patients' IgE binding to grass pollen allergens

Next, we analyzed whether rabbit antibodies induced by immunization with the various PreS fusion proteins can inhibit allergic patients' IgE binding to allergens. The relative inhibition (as a percentage) of IgE binding obtained with antibodies from rabbits immunized with different adjuvants and antigens are shown for each of the 4 grass pollen allergens in Table I. Inhibition experiments were performed with sera from patients with grass pollen allergy who were sensitized against the individual allergens (Phl p 1, n = 19; Phl p 2, n = 19; Phl p 5, n = 16; and Phl p 6, n = 21). For each wild-type allergen and PreS fusion protein, rabbits immunized with CFA and aluminum hydroxide (Table I, CFA and Alu I) as an adjuvant are displayed. Furthermore, one rabbit was immunized with the KLH-coupled peptides in CFA for comparison.

Overall, we found that the mean degree of inhibition of IgE binding was in a similar range for antibodies raised with the complete allergens, PreS fusion proteins, or KLH-coupled peptides (Table I).

The comparison of the percentage of inhibition of IgE binding obtained with anti–Phl p 6-A and anti–Phl p 6-B antibodies indicated that the blocking antibody responses induced by Phl p 6-B containing 4 peptide copies was stronger than that induced by Phl p 6-A containing only 2 peptide copies. Interestingly, the blocking antibody responses induced with the Phl p 5-D fusion protein were slightly better than those induced with the Phl p 5-C fusion protein, although only the order of the peptides in the constructs was different (Table I). Blocking antibody responses induced with the Phl p 1 and Phl p 2 variants were comparable regardless of the number of peptide copies in the constructs. However, one rabbit immunized with aluminum hydroxide–adsorbed Phl p 2-A containing only 2 peptide copies did not mount Phl p 2–specific IgG responses (see Fig E5). Regarding the Phl p 1 derivatives, we observed that Phl p 1-A adsorbed to CFA induced less blocking IgG than CFA-adsorbed Phl p 1-B.

### Selection of the final components of the grass pollen allergy vaccine BM32

Because none of the tested derivatives showed allergen-specific IgE reactivity, the best inducers of blocking IgG (ie, Phl p 6-B, Phl p 5-D, Phl p 2-B, and Phl p 1-B) were selected as components for the final vaccine. They obtained the following designations: Phl p 1-B, BM321; Phl p 2-B, BM322; Phl p 5-D, BM325; and Phl p 6-B, BM326. These components were manufactured at the scale of several hundred milligrams without a His-tag and by using a Good Manufacturing Practice compatible process (Huber, unpublished). The resulting material was applied for further evaluation of the allergenic activity, T-cell reactivity, and induction of proinflammatory cytokines.

### Mixture of recombinant BM321, BM322, BM325, and BM326 shows almost completely abolished allergenic activity

**Fig 4:**
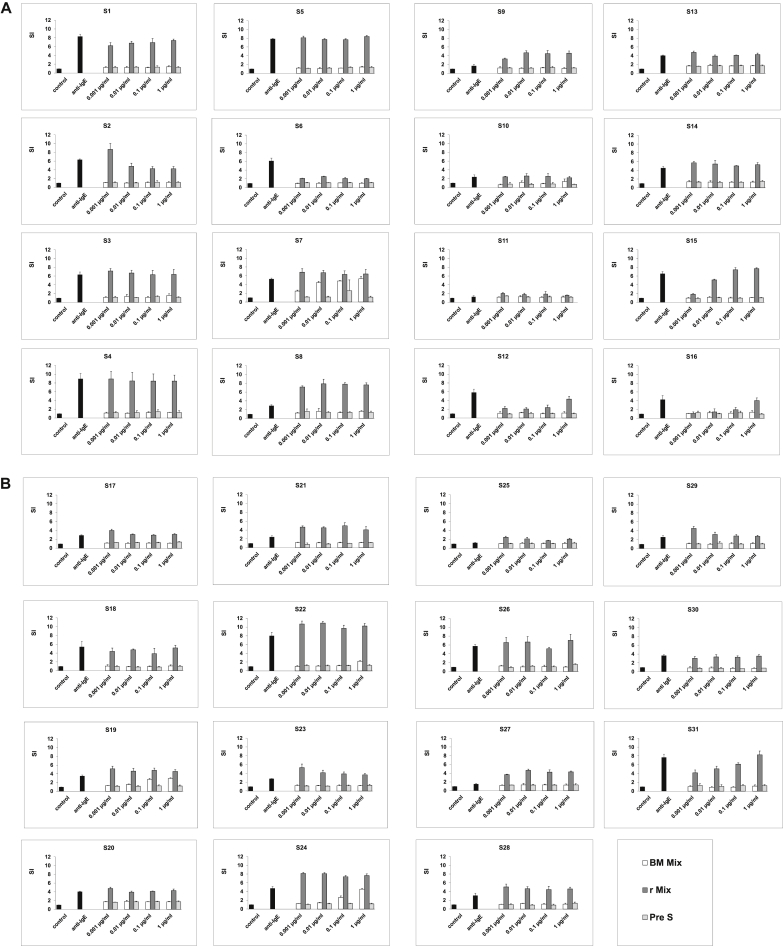
Comparison of the allergenic activity of an equimolar mix of recombinant grass pollen allergens (Phl p 1, 2, 5, and 6) with PreS and an equimolar mix of recombinant BM proteins (BM321-6). Basophils from patients with grass pollen allergy (n = 31; **A**: S1-S16; **B**: S17-S31) were exposed to different concentrations of the proteins (*x-axes*; *white bars*, BM mix; *dark gray bars*, allergen mix; *light gray bars*, PreS), anti-IgE, or buffer (controls; *black bars*). Allergen-induced upregulation of CD203c was calculated from mean fluorescence intensities (MFIs) obtained with stimulated (MFI_stim_) and unstimulated (MFI_control_) cells and is expressed as the stimulation index (MFI_stim_: MFI_control_; means ± SDs of triplicates; *y-axes*).

By using blood samples from 31 patients with grass pollen allergy, which had been collected at the grass pollen peak season of 2011, the allergenic activity of a mix of the 4 BM32 proteins (ie, BM32 mix) was compared with that of an equimolar mix of the corresponding wild-type allergens (Phl p 1, Phl p 2, Phl p 5, and Phl p 6; Fig 4). Although basophils of 23 of the 31 the patients showed full upregulation of CD203c expression already at the lowest wild-type allergen concentration tested (ie, 0.001 μg/mL), the BM32 mix did not induce any relevant upregulation of CD203c expression in basophils from 29 of the 31 patients, even at the highest concentration tested (ie, 1 μg/mL; Fig 4). Only in 2 patients (ie, S7 and S24) was a residual allergenic activity of the BM32 mix found (Fig 4).

### The BM32 mix induces lower T-cell proliferation and production of proinflammatory cytokines in cultured PBMCs from patients with grass pollen allergy than grass pollen allergens

**Fig 5:**
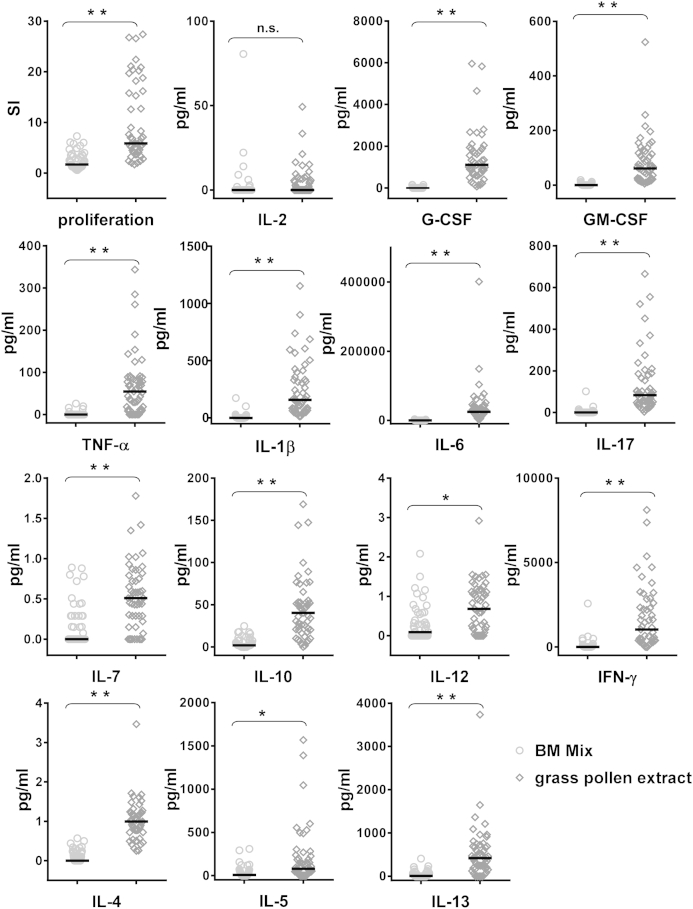
Comparison of a mix of recombinant BM proteins with natural grass pollen extract regarding the induction of proliferation and cytokine production in PBMCs of patients with grass pollen allergy. Lymphocyte proliferation responses (stimulation indices *[SI]*) and cytokine levels (in picograms per milliliter; *y-axes*) are displayed for cultured PBMCs from 50 patients with grass pollen allergy after exposure to the mix of BM proteins (left, *open circles*, 0.5 μg per well of each BM32 protein) or grass pollen extract (right, *open diamonds*, 50 μg per well of total proteins). Significant differences between the mix of BM proteins and grass pollen are indicated. *ns*, Not significant. *P < .05 and **P < .0001.

PBMCs from 50 patients with grass pollen allergy were exposed to the BM32 protein mix and natural timothy grass pollen allergens (Fig 5). We found that the BM32 mix induced a significantly lower T-cell proliferation compared with natural grass pollen allergens. There was no significant difference regarding the secretion of IL-2 in the cultures of BM32- or grass pollen allergen–stimulated PBMC cultures. However, the BM32 mix induced a significantly lower production of proinflammatory cytokines (ie, granulocyte colony-stimulating factor, GM-CSF, TNF-α, IL-1β, IL-6, IL-17, IL-7, IL-12, IFN-γ, IL-4, IL-5, and IL-13; Fig 5).

### IgG antibodies induced by means of immunization with the BM32 grass pollen vaccine inhibit allergen-induced basophil activation in patients and mice

**Fig 6:**
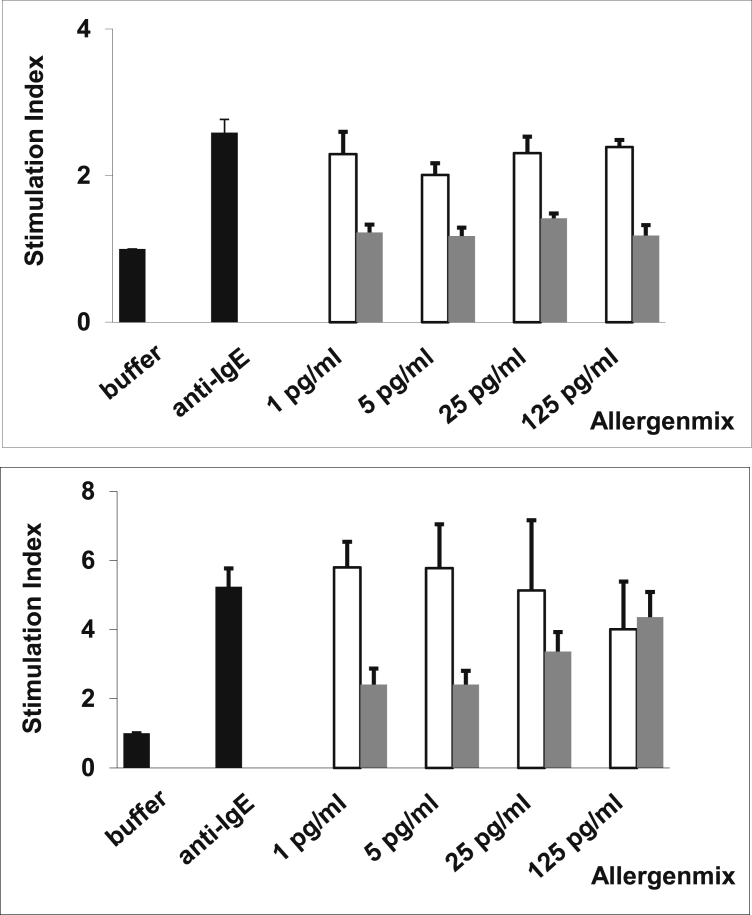
Inhibition of allergen-induced upregulation of CD203c on basophils of patients with grass pollen allergy by anti-BM32 IgG antibodies. Shown are representative results for basophils from 2 patients *(upper and lower panels)* with grass pollen allergy. Patients' PBMCs were incubated with different concentrations of the mix of recombinant grass pollen allergens that had been preincubated with rabbit anti-BM32 IgG antibodies *(gray bars)* or IgG from rabbits before immunization (Pre IgG: *white bars*), with anti-IgE or buffer alone (*black bars*; *x-axes*). Upregulations of CD203c expression are shown as mean stimulation indices ± SD *(y-axes)*.

We also immunized rabbits with an aluminum hydroxide–adsorbed mix containing 40 μg of each BM32 protein and investigated whether anti-BM32 IgG antibodies can inhibit grass pollen allergen–induced basophil activation in patients with grass pollen allergy. As exemplified for 2 patients in Fig 6, we found that incubation with anti-BM32 antibodies lead to a 25-fold to more than 100-fold inhibition of allergen-induced basophil activation compared with incubation with the corresponding preimmune antibodies.

**Fig 7:**
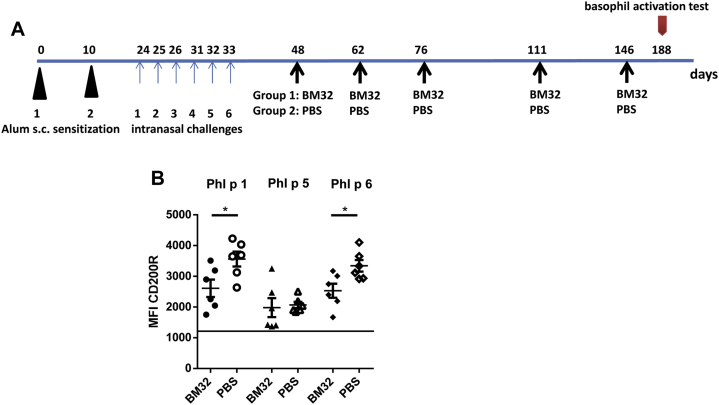
**A,** Scheme of treatment with BM32 in a murine model of grass pollen allergy. Mice were sensitized by means of subcutaneous *(s.c.)* injection and by means of intranasal challenge with recombinant grass pollen allergens split into 2 groups, one of which was treated with BM32 and the other with PBS alone. Allergen-induced basophil activation testing was done on day 188. **B,** Basophils from grass pollen–sensitized mice treated with BM32 (n = 6) or PBS (n = 6, *x-axis*) were stimulated with either Phl p 1, Phl p 5, or Phl p 6 *(top line)*. Shown are the mean fluorescence intensities (*MFIs*; *y-axis*) of CD200R expression for the mice in each group. Medians are indicated as *horizontal bars* for each group, and significant differences between the BM32 and PBS groups are indicated. *P < .05. The *horizontal line* indicates mean baseline expression of CD200R expression for the medium controls.

Similar results were obtained in an *in vivo* model of grass pollen allergy (Fig 7, *A*). Treatment with BM32 reduced allergen-induced basophil activation in sensitized mice significantly for Phl p 1 and Phl p 6 (*P* < .05) when compared with that seen in PBS-treated mice. A reduction of basophil activation was noted also for Phl p 5 stimulation (Fig 7, *B*).

## Discussion

In this study we have used the peptide-carrier technology^22,23^ to develop a hypoallergenic vaccine for SIT of grass pollen allergy. Grass pollen is one of the most potent and frequent elicitors of respiratory allergies treated with SIT. However, SIT with natural grass pollen allergens induces frequently severe side effects.^11^ With the goal to produce a safe vaccine, we have used nonallergenic peptides from the IgE-binding sites of the 4 major timothy grass pollen allergens Phl p 1, Phl p 2, Phl p 5, and Phl p 6 for construction of the vaccine. The 4 timothy grass pollen allergens were selected as templates because extensive IgE reactivity and IgE cross-reactivity studies, as well as clinical studies, indicated that they are the clinically most relevant allergens in patients with grass pollen allergy.^29^ The allergen-derived but nonallergenic peptides are fused to a carrier protein to obtain fusion proteins that, upon immunization, induce allergen-specific IgG antibodies that are focused to the major IgE-binding sites on the allergens. The induced allergen-specific IgG antibodies hence are expected to block patients' IgE binding to the allergen and the subsequent allergic immune responses caused by the formation of IgE-allergen immune complexes (eg, allergen-induced mast cell and basophil activation, IgE-facilitated allergen presentation, and allergen-induced boosts of IgE production).^7^

**Fig E6:**
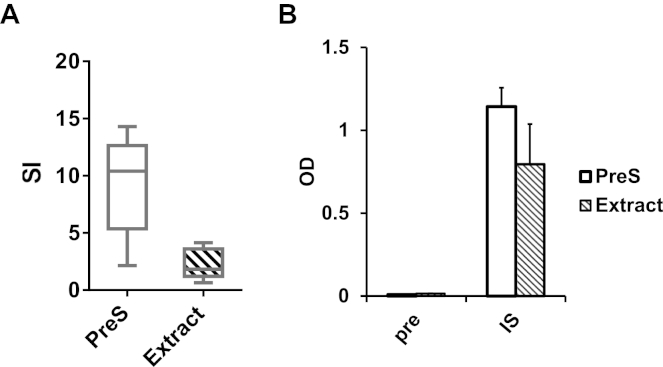
Lymphocyte proliferation and IgG_1_ responses in BM32-immunized mice. Shown are lymphocyte proliferation responses at week 22 (stimulation indices *[SI]* as box plots: *y-axis*; *whiskers* = minimum and maximum; *horizontal bars* = medians, *boxes* = 25th to 75th percentiles) to PreS and grass pollen allergen extract (*x-axis*) **(A)** and IgG_1_ reactivities in preimmune sera *(pre)* and immune sera (*IS*: week 22; median ± SD OD levels, *y-axis*) measured for 6 BM32-immunized mice **(B)**.

We were able to identify nonallergenic peptides from the IgE-binding epitopes of the 4 major timothy grass pollen allergens Phl p 1, Phl p 2, Phl p 5, and Phl p 6. By means of their incorporation into fusion proteins with PreS, we were able to obtain vaccine candidates that were easy to manufacture in large amounts. We have already described hypoallergenic fusion proteins for cat and birch pollen allergy using PreS as a carrier,^32,45^ which were obtained through a similar design process. The immunologic characterization of the BM32 fusion proteins showed that they had almost completely lost their allergenic activity, as demonstrated in extensive IgE-binding and basophil activation experiments. Furthermore, the BM32 fusion proteins showed significantly reduced ability to induce the proliferation of allergen-specific T cells and the release of proinflammatory cytokines when compared with grass pollen allergens in cultured PBMCs from allergic patients. The strongly reduced allergenic activity of the BM32 proteins opens the possibility to safely administrate high doses of the BM32 mix to allergic patients with only a few injections, which would offer significant practical advantages for their clinical use as vaccines. This assumption was supported by immunization experiments showing that the BM32 proteins induced high levels of allergen-specific IgG antibodies on immunization of rabbits. The analysis of antibody and T-cell responses in BM32-immunized mice showed that T-cell help for the production of IgG antibodies comes mainly from carrier (ie, PreS)–specific T cells (see Fig E6 in this article's Online Repository at www.jacionline.org).

Rabbit IgG antibodies induced by immunization with BM32 inhibited allergic patients' IgE binding to the wild-type allergens to a similar degree as IgG antibodies induced by immunization with wild-type allergens. Furthermore, BM32-induced IgG inhibited allergen-specific basophil activation, suggesting that a BM32-based vaccine will induce allergen-specific IgG, which protects patients against immediate allergic inflammation, the most frequent and common cause of allergic symptoms in patients with grass pollen allergy. The therapeutic effect of the BM32 vaccine could also be shown in an experimental animal model of grass pollen allergy in which treatment with BM32 reduced allergen-induced basophil activation in sensitized mice. Studies performed with natural allergen extract–based vaccines, CpG-conjugated allergens, and vaccines based on recombinant allergens and hypoallergens have shown that allergen-specific IgG also prevents boosts of allergen-specific IgE production and allergen-specific T-cell activation.^47-51^ Thus it is tempting to speculate that vaccination with BM32 will have similar beneficial effects and will also reduce allergen-specific T-cell responses and IgE production in addition to blocking allergen-induced mast cell and basophil activation. First experiences obtained with BM32 in clinical trials (skin test study: NCT01350635; safety and dose-finding study, phase IIa: NCT01445002; multicenter, double-blind, placebo-controlled efficacy study, phase IIb: NCT01538979) suggest that BM32 will bring us closer to an effective, safe, and convenient grass pollen allergy vaccine, which might be beneficial for a large number of allergic patients.

**Clinical implications**

The recombinant hypoallergenic allergy vaccine BM32 reported in our study should allow safe and effective immunotherapy of grass pollen allergy.

## Figures and Tables

**Table E1:** Characteristics of amino acids and molecular weights of synthetic allergen peptides

Allergen	Accession no.	Amino acid position	Sequence	No. of amino acids	Weight (Da)
Phl p 1	P43213				
P5-1		212-241	**C**VRYTTEGGTKTEAEDVIPEGWKADTSYESK	31	3452
Phl p 2	P43214				
P1-2		1-33	VPKVTFTVEKGSNEKHLAVLVKYEGDTMAEVEL**C**	34	3765
P2-2		8-39	VEKGSNEKHLAVLVKYEGDTMAEVELREHGSD**C**	33	3674
P3-2		34-64	REHGSDEWVAMTKGEGGVWTFDSEEPLQGPFN**C**	33	3697
P4-2		66-96	**C**FRFLTEKGMKNVFDDVVPEKYTIGATYAPEE	32	3699
Phl p 5	Q40960				
P1-5		26-58	ADLGYGPATPAAPAAGYTPATPAAPAEAAPAGK**C**	34	3068
P2-5		59-92	**C**ATTEEQKLIEKINAGFKAALAAAAGVQPADKYR	34	3578
P5-5		176-212	**C**AEEVKVIPAGELQVIEKVDAAFKVAATAANAAPANDK	38	3853
P6-5		217-246	**C**EAAFNDAIKASTGGAYESYKFIPALEAAVK	31	3236
Phl p 6	Y16956.1				
P1-6		23-54	GKATTEEQKLIEDVNASFRAAMATTANVPPAD**C**	34	3451
P2-6		56-90	**C**YKTFEAAFTVSSKRNLADAVSKAPQLVPKLDEVYN	36	4005
P3-6		73-104	**C**DAVSKAPQLVPKLDEVYNAAYNAADHAAPEDKY	34	3678
P4-6		95-127	**C**AADHAAPEDKYEAFVLHFSEALRIIAGTPEVHA	34	3661

For each allergen (Phl p 1, Phl p 2, Phl p 5, and Phl p 6), peptides of different locations and lengths were synthesized. Displayed are the names of allergens and peptides, database accession numbers of allergens, positions of peptides in the allergens, sequences (cysteines added to facilitate coupling are shown in boldface and underlined), numbers of amino acids, and molecular weights (in daltons).

**Table E2:** Inhibition of allergic patients' IgE binding to Phl p 2 with IgG specific for rPhl p 2 or for Phl p 2 peptides (P1-2 to P4-2)

Patient no.	OD	rPhl p 2 (%)	P1-2 (%)	P2-2 (%)	P3-2 (%)	P4-2 (%)
1	0.877	8.37	3.26	48.41	60.93	75.97
2	0.986	12.15	5.89	51.18	72.33	72.71
3	1.470	5.99	0.17	25.29	49.55	56.66
4	1.492	10.90	0.00	41.21	55.30	65.27
5	0.826	10.53	0.00	34.95	49.53	66.43
6	1.048	13.58	2.78	50.48	50.11	70.14
7	1.290	0.41	0.00	44.73	51.72	68.80
8	1.095	5.30	0.00	32.98	49.05	65.08
9	0.960	5.86	5.08	28.71	37.66	57.55
10	0.902	0.00	1.06	18.89	25.46	44.39
11	0.597	5.80	2.54	39.59	43.23	68.33
12	0.715	12.11	1.31	25.68	34.67	65.95
13	1.380	14.23	3.97	56.51	68.18	83.39
14	0.647	33.16	0.00	87.63	88.93	92.59
15	0.683	18.44	2.19	71.95	75.27	87.91
16	1.542	8.32	1.63	68.80	76.19	89.21
17	0.527	12.07	2.43	68.54	71.99	91.60
18	0.651	19.06	2.84	78.57	78.59	91.10
19	1.567	5.96	3.72	26.27	25.31	71.06
20	1.027	21.09	10.55	42.94	42.10	76.76
21	1.155	7.66	0.00	25.32	30.12	80.23
Mean	1.02	11.00	2.35	46.13	54.11	73.39

Displayed are Phl p 2–specific IgE levels (OD levels: first column, noninhibited) for 21 patients and the percentages of inhibition of IgE binding after preincubation with allergen- or peptide-specific IgG. Mean values are shown in the bottom line.

**Table E3:** Inhibition of allergic patients' IgE binding to Phl p 6 with IgG specific for rPhl p 6 or for Phl p 6 peptides (P1-6 to P4-6)

Patient no.	OD	rPhl p 6 (%)	P1-6 (%)	P2-6 (%)	P3-6 (%)	P4-6 (%)
1	0.304	80.14	81.69	73.38	10.17	63.26
2	0.650	92.92	86.14	91.53	1.36	41.12
3	0.455	85.95	88.58	87.37	27.43	59.84
4	0.450	67.74	66.10	63.30	25.99	36.67
5	0.332	74.02	61.99	53.38	18.54	27.29
6	0.719	70.61	73.46	65.03	13.00	53.39
7	0.279	75.96	76.78	67.51	36.49	67.96
8	0.276	41.22	34.88	7.99	31.30	19.01
9	1.669	93.29	88.16	86.06	44.83	71.19
10	1.913	85.96	80.94	85.75	0.00	21.04
11	0.258	66.75	47.34	57.04	12.25	22.89
12	0.123	45.17	61.05	29.00	27.42	43.47
13	0.275	40.27	47.92	43.45	0.00	30.86
14	0.110	94.34	93.55	93.01	27.34	67.21
15	2.014	83.13	68.82	73.71	35.92	61.12
Mean	0.65	73.17	70.50	65.17	20.80	45.75

Displayed are Phl p 6–specific IgE levels (OD levels: first column, noninhibited) for 15 patients and percentages of inhibition of IgE binding after preincubation with allergen- or peptide-specific IgG. Mean values are shown in the bottom line.

**Table I:** Percentages of inhibition of allergic patients' IgE binding to allergens with specific rabbit IgG antibodies

Allergen	CFA	Alu I	CFA	Alu I	CFA	Alu I	CFA	No. of sera
Phl p 1								
Immunogen	Phl p 1		Phl p 1-A		Phl p 1-B		Peptide-KLH	
Mean	95.1	93.0	42.2	88.7	91.7	68.7	94.3	n = 19
Range	90.3-97.9	89.6-96.6	10.7-66.6	75-93.2	77.9-96.5	36.2-76.5	81.7-97.8	
Phl p 2								
Immunogen	Phl p 2		Phl p 2-A		Phl p 2-B		Peptide-KLH	
Mean	87.8	89.5	94.0	91.4	89.0	91.0	83.2	n = 19
Range	62.4-95	61.8-96.7	89.0-97.3	86.3-95.8	77.9-90.02	80.2-94.1	54.5-91.3	
Phl p 5								
Immunogen	Phl p 5		Phl p 5-C		Phl p 5-D		Peptide-KLH	
Mean	97.0	95.3	73.8	82.4	82.3	92.1	79.3	n = 16
Range	90.9-99.5	89.7-97.3	54.2-89.7	76.8-92	71.9-92.5	84.1-96.7	60.9-92.9	
Phl p 6								
Immunogen	Phl p 6		Phl p 6-A		Phl p 6-B		Peptide-KLH	
Mean	81.4	46.5	84.1	46.0	83.4	67.0	82.8	n = 21
Range	78-99	24.9-87.2	17.2-96.3	21.8-78.2	49.1-96.5	20.2-75.7	44.9-97.4	

*Alu I*, Aluminum hydroxide.

## Methods

### Allergic patients' sera and blood samples, timothy grass pollen extract, recombinant allergens, synthetic peptides, and antibodies

Patients with grass pollen allergy, as well as nonallergic control subjects, were characterized by case history and results of skin prick testing and measurement of specific IgE antibodies to recombinant grass pollen allergens (rPhl p 1, rPhl p 2, rPhl p 5, and rPhl p 6), as previously described.^E1^ Anonymized residual serum samples from these patients and from subjects without grass pollen allergy were used for serologic experiments with approval of the ethics committee of the Medical University of Vienna.

Heparinized blood samples for T-cell proliferation and CD203c assays were obtained from patients with grass pollen allergy (NCT01350635) after informed consent was provided.

Recombinant grass pollen allergens were provided by Biomay AG. Timothy grass pollen extract was obtained from Stallergenes. Phl p 2 and Phl p 6 peptides were synthesized and purified, as previously described.^E2^ Rabbit antibodies specific for allergens (Phl p 1, Phl p 2, Phl p 5, and Phl p 6) or allergen-derived peptides were obtained by immunizing rabbits with purified recombinant proteins or with the KLH-coupled peptides (Charles River, Kissleg, Germany) 3 times in monthly intervals with 200 μg of antigen emulsified once in CFA and twice in Freund incomplete adjuvant. Antibodies specific for PreS fusion proteins were raised by using the same CFA protocol and additionally by immunizing with aluminum hydroxide–adsorbed proteins (100 μg per injection for single fusion proteins or with the BM32 vaccine containing 40 μg of each of the 4 fusion proteins). PreS-specific rabbit antibodies are described by Niespodziana et al.^E3^

### Purification of His-tagged recombinant PreS and peptide-carrier fusion proteins

For Phl p 1-A and Phl p 1-B, Phl p 6-A and Phl p 6-B, or Phl p 5-A, Phl p 5-B, and Phl p 5-D, the bacterial cell pellets obtained from induced liquid cultures were thawed for 15 minutes on ice and resuspended under native conditions in lysis buffer I (50 mmol/L NaH_2_PO_4_ and 200 mmol/L NaCl containing 10 mmol/L imidazole, pH 7.5). After incubation with lysozyme (1 mg/mL, Sigma-Aldrich) for 30 minutes on ice, cells were lysed by means of repetitive freezing and thawing, and DNAse I (5 μg/mL) was added. For PreS, Phl p 2-A, Phl p 2-B, and Phl p 5-C cell pellets were thawed for 15 minutes on ice and solubilized in Buffer II (100 mmol/L NaH_2_PO_4_ and 10 mmol/L Tris-HCl, pH 8.0, containing 6 mol/L Gu HCl for PreS or 8 mol/L urea for Phl p 2-A, Phl p 2-B, and Phl p 5-C) by stirring for 1 hour at room temperature.

In both protocols the lysates were cleared by means of centrifugation (14,000 rpm for 20 minutes at 4°C), and recombinant fusion proteins were purified by using nickel affinity chromatography (Qiagen, Hilden, Germany). For proteins from inclusion bodies, elution was done with buffer II containing 8 mol/L urea (pH 3.5). Urea was removed by using dialysis either against 10 mmol/L Tris (pH 7.5) for PreS fusion proteins or against 10 mmol/L NaH_2_PO_4_ (pH 4.8) for PreS. For purification under native conditions, an elution buffer containing 10 mmol/L Tris, 200 nmol/L NaCl, and 250 mmol/L imidazole was used, followed by ultrafiltration (Amicon Ultra 15; Merck KGaA, Darmstadt, Germany) for concentration of samples. The purity of recombinant proteins was analyzed by using SDS-PAGE and Coomassie staining. Protein concentrations were determined by measuring protein-specific ODs as 280 nm^E4^ and by using a BCA Protein Assay Kit (Novagen, Madison, Wis).

The identity of the fusion proteins was confirmed by means of immunoblotting with rabbit anti-peptide anti-sera (Phl p 1: rabbit anti-P5; Phl p 2: rabbit anti-P4; Phl p 6: rabbit anti-P1; Phl p 5: rabbit anti-P1, anti-P2, anti-P5, and anti-P6) and anti-PreS antibodies diluted 1:2500.^E3^ Bound rabbit antibodies were detected with iodine 125–labeled goat anti-rabbit IgG (PerkinElmer, Waltham, Mass) diluted 1:2500 for 1 hour and visualized by means of autoradiography.^E5^

### Mouse immunization, antibody, and T-cell proliferation responses

Six-week-old female BALB/c mice (Charles River, Sulzfeld, Germany) were used in the study. The animals were maintained in the Animal Care Unit of the Department of Pathophysiology and Allergy Research (Medical University of Vienna), according to local guidelines for animal care. Six mice were immunized subcutaneously 3 times with BM32, containing 10 μg of each BM32 component adsorbed to aluminum hydroxide (SERVA Electrophoresis, Heidelberg, Germany) in 4-week intervals and once after 16 weeks. Blood samples were drawn from the tail vein before immunization at weeks 4, 8, 12, 16, and 22. Lymphoproliferative responses in mice were measured in spleen cell cultures at week 22 stimulated with PreS (0.06 μg per well) and timothy grass pollen extract (10 μg per well, Stallergenes), concanavalin A (0.5 μg per well, positive control), or medium alone, as previously described.^E6^ PreS and grass pollen allergen extract–specific IgG_1_ responses were measured by using ELISA.^E6^ Results from T-cell experiments and IgG_1_ measurements are shown as medians ± SDs for the group of 6 mice.

### Mouse model for grass pollen allergy, treatment with BM32, and basophil activation testing

Female BALB/c mice aged 6 to 8 weeks (Charles River) were maintained at the animal facility of the University of Salzburg, according to local guidelines. Mice (n = 12) were sensitized on days 0 and 10 by means of subcutaneous injection with a mixture of Phl p 1 (5 μg), Phl p 5 (1 μg), and Phl p 6 (5 μg) adsorbed to aluminum hydroxide (Alu-Gel, SERVA). On days 24, 25, 26, 31, 32, and 33, mice were boosted by means of intranasal application of a mixture containing 5 μg of Phl p 1, 1 μg of Phl p 5, and 5 μg of Phl p 6 in 40 μL of PBS. Phl p 2 was not included because of its poor allergenicity and immunogenicity in mice.^E7^ One group of mice (n = 6) was treated on days 48, 62, 76, 111, and 146 by means of subcutaneous injection of 200 μL of alum-adsorbed BM32 (containing 20 μg of each component), whereas the control group (n = 6) was sham treated by means of subcutaneous injection of 200 μL of PBS. The basophil activation test was done at day 188. For this purpose, heparinized blood samples from mice were diluted in RPMI and incubated with allergens (30 μL of blood was diluted with 30 μL of RPMI 1640 [PAA, Pasching, Austria] containing 200 μg/mL heparin and 100 ng/mL Phl p 1, 4 ng/mL Phl p 5, or 4 ng/mL Phl p 6) for 2 hours at 37°C and 7% CO_2_ in 96-well V-bottom plates in duplicates (Greiner, Kremsmünster, Austria). Cells were washed with PBS, 1% wt/vol BSA, and 2 mmol/L EDTA and centrifuged at 500*g* for 5 minutes before surface staining with fluorescein isothiocyanate anti-mouse IgE (RME-1; BioLegend, San Diego, Calif), peridinin-chlorophyll-protein complex/Cy5.5 anti-mouse CD4 (GK1.5, BioLegend), phycoerythrin/Cy7 anti-mouse CD19 (6D5, BioLegend), and anti-CD200R (OX110; eBioscience, San Diego, Calif), all diluted 1:200 in PBS, 1% wt/vol BSA, and 2 mmol/L EDTA. After incubation, cells were washed twice with PBS, 1% wt/vol BSA, and 2 mmol/L EDTA, followed by erythrocyte lysis through resuspending the pellet in 100 μL of FACS Lysing Solution (Becton Dickinson, San Jose, Calif). After a final wash, cells were analyzed on a FACSCanto II flow cytometer (Becton Dickinson). T cells (CD4^+^) and B cells (CD19^+^) were gated out, and basophils were gated as IgE^+^ and CD200R^+^ cells.^E8^ Mean fluorescence intensities with an error of less than 10% are shown.
